# Complete genome sequence of Halomonas sp. R5-57

**DOI:** 10.1186/s40793-016-0192-4

**Published:** 2016-09-07

**Authors:** Adele Williamson, Concetta De Santi, Bjørn Altermark, Christian Karlsen, Erik Hjerde

**Affiliations:** 1 Department of Chemistry, UiT- The Arctic University of Norway, N-9019 Tromsø, Norway; 2 Division of Aquaculture, Nofima AS, PO Box 210, Ås, N-1431 Norway

**Keywords:** *Halomonas*, Growth temperature, Salt tolerance, Secreted enzymes, Osmolyte, Polyhydroxyalkanoates

## Abstract

The marine Arctic isolate *Halomonas* sp. R5-57 was sequenced as part of a bioprospecting project which aims to discover novel enzymes and organisms from low-temperature environments, with potential uses in biotechnological applications. Phenotypically, *Halomonas* sp. R5-57 exhibits high salt tolerance over a wide range of temperatures and has extra-cellular hydrolytic activities with several substrates, indicating it secretes enzymes which may function in high salinity conditions. Genome sequencing identified the genes involved in the biosynthesis of the osmoprotectant ectoine, which has applications in food processing and pharmacy, as well as those involved in production of polyhydroxyalkanoates, which can serve as precursors to bioplastics. The percentage identity of these biosynthetic genes from *Halomonas* sp. R5-57 and current production strains varies between 99 % for some to 69 % for others, thus it is plausible that R5-57 may have a different production capacity to currently used strains, or that in the case of PHAs, the properties of the final product may vary. Here we present the finished genome sequence (LN813019) of *Halomonas* sp. R5-57 which will facilitate exploitation of this bacterium; either as a whole-cell production host, or by recombinant expression of its individual enzymes.

**Electronic supplementary material:**

The online version of this article (doi:10.1186/s40793-016-0192-4) contains supplementary material, which is available to authorized users.

## Introduction

*Halomonas* sp. R5-57 is a marine member of the *Halomonadaceae*, a family of Gram-negative chemoorganotrophic bacteria that display moderate to high salt tolerance. Members of this genus have been isolated from diverse saline environments such as ocean water [1, 2], salterns [3], marine hydrothermal vents [4], hypersaline lakes [5, 6] and salted fermented food [7]. Several species of *Halomonas* have also been identified as human pathogens [1, 8, 9]. To date draft genomes of 15 *Halomonas* species (*H. zincidurans* B6, *H. halodenitrificans*DSM 735, DSM 1457, *H. lutea*DSM 2350, *H. anticariensis* FP35 DSM 16096, *H. zhanjiangensis*DSM 2107, *H. jeotgali* Hwa, *H. titanicae* BH1, *H. smyrnensis* AAD6, *H. stevensii*S18214, *H. boliviensis* LC1, *H. caseinilytica* ASM81542v1, *H. hydrothermalis* HaloHydro1.0, *H. xinjiangensis* ASM75934v1 and *H. salina*) and complete genomes of two species (*H. elongata*DSM 2581 ASM19687v1 and *H. campaniensis* ASM69648v1) are available.

*Halomonas* species have a number of technologically exploitable features. Both compatible solutes, which the bacteria accumulate as part of their adaptation to saline environments, and extracellular polymers, which protect the cells from environmental stresses and aid in biofilm formation, are used in pharmaceutical, food-processing and biotechnological industries [10, 11]. Additionally, polyhydroxyalkanoates which are accumulated by the bacterium as energy storage compounds can be used to produce biodegradable plastic materials [12]. Finally, the high solubility of *Halomonas* proteins, both in their folded and unfolded states have led to their use as fusion tags for improving the solubility of recombinantly expressed proteins [13].

The isolation, characterization and genome sequencing of *Halomonas* sp. R5-57 was undertaken as part of the MARZymes project which aims to identify novel cold-adapted enzymes and organisms from marine sources. Here we present the complete genome sequence of *Halomonas* sp. R5-57 together with its temperature and salinity growth optima and functional screening for various activities.

## Organism information

### Classification and features

*Halomonas* sp. R5-57 was isolated from the skin of the red sea squirt *Halocynthia papillosa* collected from the Barents Sea in Spring 2009. The animal was dissected and the skin homogenized in an equal volume of sterile sea water and 50 μl was plated onto IM8 media [14]. An individual colony was picked from this raw plate after incubation at 4 °C for two weeks, and was subsequently re-streaked two times and grown at 4 °C for 1 week. Liquid cultures for DNA isolation and growth curves were prepared by inoculating Luria-Bertani media with 3.5 % NaCl from these pure isolates. A summary of the isolation and phenotypic characteristics of *Halomonas* sp. R5-57 are given in Table 1.

**Table 1:** Classification and general features of *Halomonas sp.* R5-57 [18]

MIGS ID	Property	Term	Evidence code^a^
	Classification	Domain: *Bacteria*	TAS [31]
		Phylum: *Proteobacteria*	TAS [32]
		Class: *Gammaproteobacteria*	TAS [33]
		Order: *Oceanospirillales*	TAS [34–36]
		Family: *Halomonadaceae*	TAS [33, 37–39]
		Genus: *Halomonas*	TAS [2, 40, 41]
		Species: *Halomonas sp.*	TAS [2, 40, 41]
		Strain: R5-57	
	Gram stain	Negative	TAS [42]
	Cell shape	Rods	IDA
	Motility	Motile	TAS [43]
	Sporulation	Not reported	NAS
	Temperature range	4 – 41 °C	IDA
	Optimum temperature	20 °C	IDA
	pH range; Optimum	8.0-10.0	TAS [43]
	Carbon source	Glucose, mannitol, inositol sorbitol, sucrose, melibiose, amygdaline, arabinose, manose, mannitol, N-acetyl glucosamine, maltose, potassium gluconate, capric acid, adipic acid malate	IDA
MIGS-6	Habitat	Marine Arctic	IDA
MIGS-6.3	Salinity	Requires >1 % NaCl, tolerates up to 12 % NaCl. Optimum is 3.5-7.0 % NaCl	IDA
MIGS-22	Oxygen requirement	Aerobic	TAS [43]
MIGS-15	Biotic relationship	Free living, isolated from the skin of the red sea squirt *Halocynthia papillosa*	NAS/IDA
MIGS-14	Pathogenicity	Not reported	NAS
MIGS-4	Geographic location	Sagaskjær	IDA
MIGS-5	Sample collection	14.05.2009	IDA
MIGS-4.1	Latitude	78.12.78372 N,	IDA
MIGS-4.2	Longitude	013.58.27000 E	IDA
MIGS-4.4	Altitude	−180.42 m	IDA

^a^Evidence codes - IDA: Inferred from Direct Assay; TAS: Traceable Author Statement (i.e., a direct report exists in the literature); NAS: Non-traceable Author Statement (i.e., not directly observed for the living, isolated sample, but based on a generally accepted property for the species, or anecdotal evidence). These evidence codes are from the Gene Ontology project [44]

**Fig. 1:**
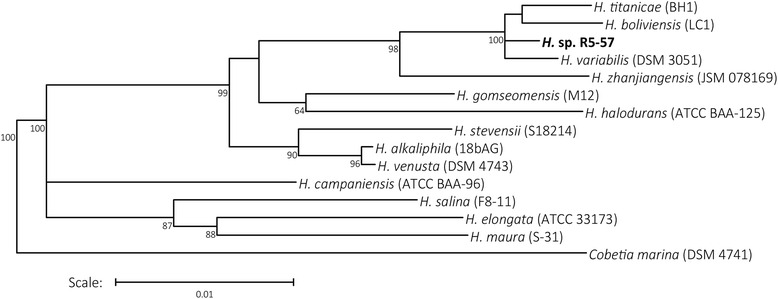
Neighbor-joining analysis of the 16S rRNA gene showing the evolutionary distance of *Halomonas* sp. R57-5 to a selection of *Halomonas* species: *H. titanicae* (BH1) (NR_117300.1), *H. boliviensis* (LC1) (NR_029080.1), *H. variabilis* (DSM 3051) (NR_042068.1), *H. zhanjiangensis* (JSM 078169) (NR_104283.1), *H. gomseomensis* (M12) (NR_042488.1), *H. halodurans* (ATCC BAA-125) (HQ449183.1), *H. stevensii* (S18214) (NR_115088.1), *H. alkaliphila* (18bAG) (NR_042256.1), *H. venusta* (DSM 4743) (NR_118033.1), *H. campaniensis* (ATTCC BAA-96) (AJ515365.2), *H. salina* (F8-11) (AJ295145.1) and *H. maura* (S-31) (NR_042010.1). Bootstrap values greater than 50 % based on 1000 repetitions are shown with *Cobetia marina* (NR_042065.1) used as an outgroup. The tree was produced using the Ribosomal Database Project (RDP) ‘Tree builder’ tool [45]: The scale bar on the tree represents the percentage sequence dissimilarity between two sequences

PCR product of the partial 16s rRNA gene was generated using the 27F and 1492R universal primers [15], and then sequenced with the BigDye terminator kit version 3.1 (Applied Biosystems) using the 515 FD primer. This placed isolate R5-57 with other psychrotolerant species of *Halomonas*, having 99 % identity to *H. glaciei* DD 39T (MTCC 4321; JCM 11692), isolated from fast ice in Antarctica [16]. Neighbor-joining analysis of the full-length 16S rRNA gene shown in Fig. 1, separates *Halomonas* sp. R5-57 from the related *H. titanicae* BH1 (99.4 %), *H. boliviensis* (99.0 %) and *H. variabilis*DSM 3051 (99.5 %).

**Fig. 2:**
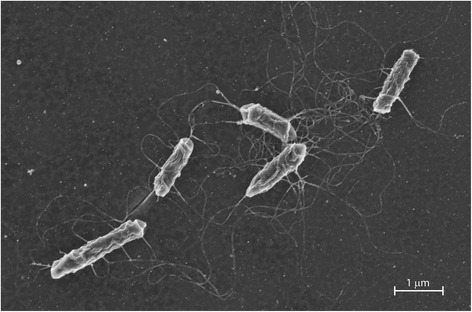
Scanning electron micrograph of *Halomonas* sp. R5-57. See main text for sample preparation

Scanning electron micrographs show that this bacterium is rod-shaped and has a number of flagella with a peritrichous arrangement (Fig. 2). Cells for microscopy were taken from colonies after 24 h growth and fixed with 5 % glutaraldehyde for 1 h, then 2.5 % glutaraldehyde overnight. Fixed suspensions were applied to Poly-L-Lysine coated slides for 2–5 min and post-fixed with 1 % osmium tetroxide for 30 min followed by dehydration with increasing concentrations of ethanol (30 %, 60 %, 90 %, 96 %, 5 min each, 99 % 5 min twice) hexamethyldisilazine (2 min, two times), and finally incubation in a dessicator with silica gel for approximately 2 h. Dried specimens were sputter-coated with gold and observed with a ZEISS MERLIN Scanning Electron Microscope with an accelerating voltage of 2.0 kV.

Members of the *Halomonadaceae* are characterized by having high salt tolerance, and as the 16S rRNA sequence of *Halomonas* sp. R5-57 clusters with other psychrotolerant strains *H. titanicae*, *H. variabilis* and *H. boliviensis**,* we investigated both the salinity and temperature optimum of this isolate. Growth rates measured on LB medium containing 0.5 - 12 % NaCl at temperatures between 4 – 41 °C show *Halomonas* sp. R5-57 has an optimum of 20 °C in 3.5 % NaCl, the salinity of seawater, and requires minimum salt concentration of 1.0 % for any significant growth to occur. The salinity of the medium also had a marked effect on the temperature tolerance of *Halomonas* sp. R5-57 as below 7 % NaCl growth rates peaked at 20 °C then decreased rapidly; but at 10 – 12 % NaCl the temperature optimum increased to 30 °C and growth was observed at up to 41 °C (Additional file 1: Figure S1).

Metabolic activities of *Halomonas* sp. R5-57 were determined with the API® system, using tests NE and E (bioMérieux). Tests were conducted at 25 °C, all media was supplemented with 3.5 % NaCl and final results were scored after 5 days. *Halomonas* sp. R5-57 is oxidase positive, reduced nitrate to nitrite, was able to utilize citrate, ferment or oxidize glucose, manitol, inositol, sorbitol, melbiose, sacharose, melibiose amygdaline arabinose, and assimilate N-acetyl glucosamine, potassium gluconate, capric acid and adipic acid. Additionally this strain displayed beta galactosidase, arginine dehydrolase gelatinase activities, and hydrolysed esculin.

Substrate utilisation was also examined by plate-based screens conducted at 4 and 20 °C on marine broth supplemented with the following indicator substrates: 1.5 % w/v carboxylmethylcellulose (cellulase); 0.1 % w/v sodium alginate (alginate lyase); 2 % w/v starch, then stained with 0.5 % Congo Red, 5 % ethanol (amylase); 2.5 g/L xylan (xylanase); 0.5 % w/v chitin (chitinase); 1 % w/v skimmed milk (protease), 0.4 % w/v gelatin then stained with Coomassie Blue G-250 (gelatinase); 1 % v/v tributyrin (lipase/esterase); or on LB media supplemented with 3.5 % NaCl and DNA (DNAse). Results were recorded by the presence of a halo on the plate after 1 week, and revealed that *Halomonas* sp. R5-57 has secreted chitinase, DNAse and protease activities at 20 °C, and lipase activity at 4 °C.

## Genome sequencing information

### Genome project history

*Halomonas* sp. R5-57 was selected for genome sequencing on the basis of its phylogenetic position that grouped this isolate with other psychrotolerant species of *Halomonas*. The project commenced with collection of the isolate in 2009, and Illumina sequencing was completed at the Norwegian Sequencing Centre in July 2012, followed by Pacific Biosciences (PacBio) sequencing in January 2015.The finished sequence of *Halomonas* sp. R5-57 was completed in February 2015 and deposited in the *European Nucleotide Archive* [17] with the identifier LN813019 (GI:802125597).

Table 2 presents the project information and its association with MIGS version 2.0 compliance [18].

**Table 2:** Project information

MIGS ID	Property	Term
MIGS 31	Finishing quality	Finished
MIGS-28	Libraries used	One Illumina Paired-End library, one 20 kb PacBio library
MIGS 29	Sequencing platforms	Illumina HiSeq 2000, Pacific Biosciences PacBio RS II
MIGS 31.2	Fold coverage	Illumina (512 ×), PacBio (16 ×)
MIGS 30	Assemblers	Mira hybrid assembly
MIGS 32	Gene calling method	Glimmer 3
	Locus Tag	HALO
	Genbank ID	LN813019
	GenBank Date of Release	Mar. 31, 2015
	GOLD ID	Gs0114368
	BIOPROJECT	PRJEB8412
MIGS 13	Source Material Identifier	The skin of the red sea squirt *Halocynthia papillosa* collected from the Barents Sea
	Project relevance	Biotechnological

### Growth conditions and genomic DNA preparation

Pure cultures of *Halomonas* sp. R5-57 were grown for two days at 20 °C to stationary phase. Growth media was in LB supplemented with 3.5 % NaCl. High molecular weight DNA was isolated using the GenElute Bacterial Genomic Kit (Sigma) following the manufacturer’s instructions for Gram negative strains. Briefly, cells were harvested by centrifugation from 1.5 ml culture, lysed in ‘*Lysis solution T’* containing RNase A followed by treatment with Protinase K. All subsequent steps involving binding to, and elution from spin columns were carried out according to the kit protocol, and the final genomic DNA sample was eluted in distilled water. Where mixing was required, gentle inversion of the sample was used in lieu of vortexing or pipetting to avoid shearing of the sample DNA. The DNA concentration was estimated by the absorbance at 260 nm, and purity was assessed by the ratio of absorbance at 260 to 280 nm measured on a Nanodrop spectrophotometer (Thermo scientific).

Genomic DNA was further prepared for Illumina sequencing by sonication using a Covaris sonicator down to ~700 bp, and the library was produced with Solid Phase Reversible Immobilization works technology (Beckman Coulter). The sample was then separated on a 2 % agarose gel (120V, 40 min) and DNA of 750-850 bp was retrieved. Afterwards PCR was performed to amplify the library.

### Genome sequencing and assembly

Sequencing of *Halomonas* sp. R5-57 used a combination of Illumina and PacBio Single Molecule Real-Time (SMRT) sequencing technology methods. Illumina sequencing (100 bp paired end) was done on a HiSeq2000 using TruSeq SBS v3 reagents (Illumina). This was followed by preparation of a PacBio library which was sequenced on the Pacific Biosciences PacBio RS II sequencer using P4-C2 chemistry [19]. The Illumina sequencing produced 26,184,828 raw reads (2,3921,979 reads after removal of artifacts) giving an average genome coverage of 512 ×, and PacBio produced 10,611 raw reads (10,460 quality filtered) with a coverage of 16 ×. The reads were assembled using MIRA hybrid assembly [20] which allowed mapping of the Illumina reads onto the PacBio scaffold for correction of indels, resulting in a single circular chromosome with no plasmids.

### Genome annotation

Genes were identified using Glimmer 3 [21] and annotated using an in-house annotation pipeline where protein-coding sequences were searched against the COG database [22] and assigned with COG numbers, signal peptides were predicted using Phobius [23], and tRNA genes were identified using the tRNAscan-SE tool [24].

## Genome properties

**Fig. 3:**
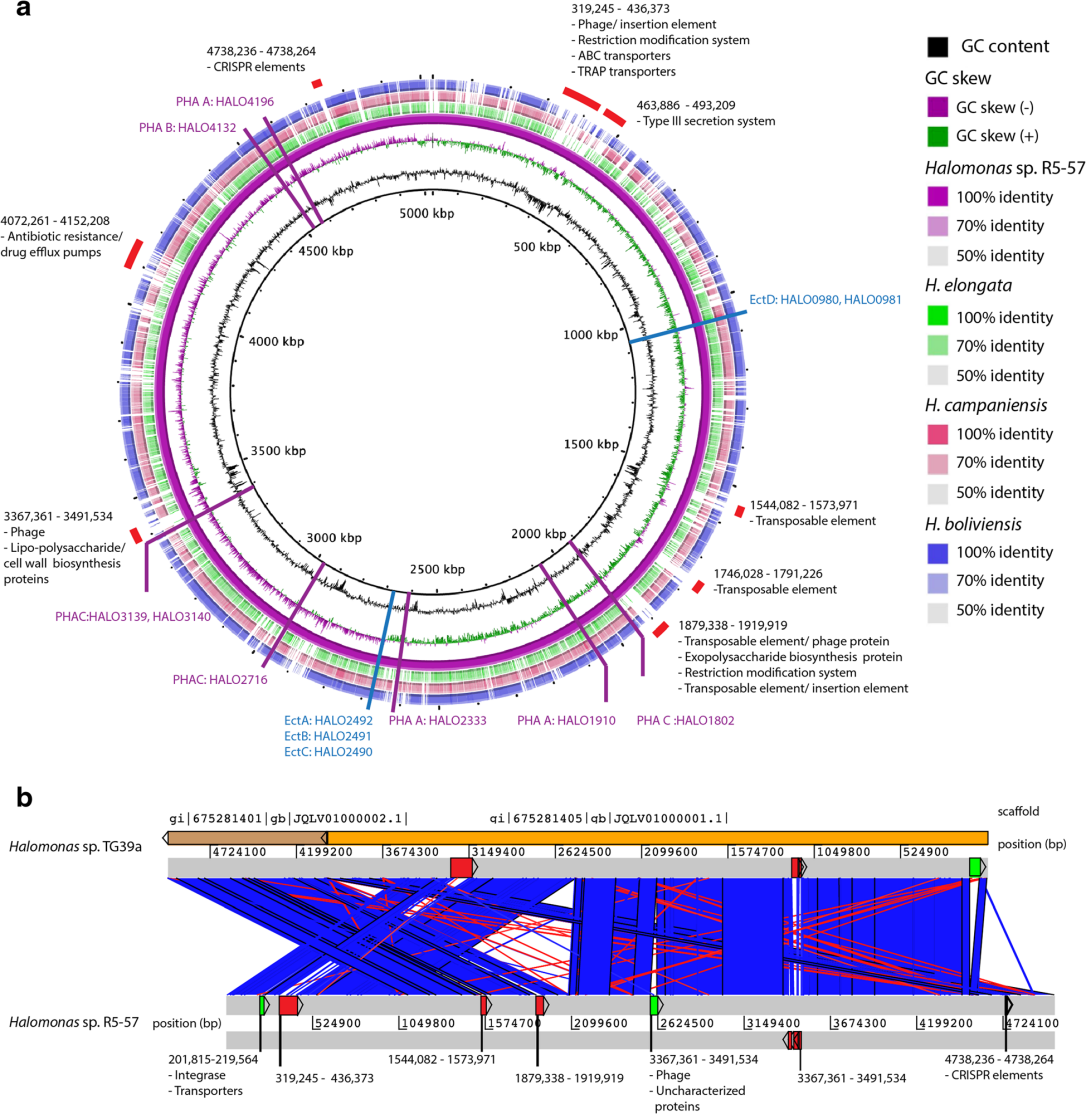
**a** Graphical representation of the 5.03 Mb chromosome of *Halomonas* sp. R5-57 indicating from innermost ring: distribution of the GC content (*black*), GC skew (*purple*/*green*), homology with self (*solid purple*), *H. elongata* DSM 2581 ASM19687v1 (*green*) *H. campaniensis* ASM69648v1 (*pink*), and *H. boliviensis* LC1 (*blue*). The outermost red blocks indicate areas where *Halomonas* sp. R5-57 has low homology with other species, and are annotated with possible genes of interest. The approximate position and locus tag of genes involved in ectoine biosynthesis are marked in blue, those producing PHA are in magenta. **b** Comparison between *Halomonas* sp. R5-57 and *Halomonas sp*. TG39a. Low homology regions which have equivalent in part A are shown in red blocks with the position numbers of the *Halomonas* sp. R5-57 - those not identified in A are shown in green and also include possible genes of interest

The genome comprises one circular chromosome of 5031571 bp which is graphically represented in Fig. 3a indicating the GC distribution (55.75 % overall) and GC skew. The properties and statistics of the genome are summarized in Tables 3 and 4. Four thousand six hundred seventy seven genes were predicted, 4599 of which are protein coding genes. Four thousand two hundred twenty five (91.87 %) of the protein coding genes were assigned to a putative function with the remaining genes annotated as hypothetical proteins.

**Table 3:** Genome statistics

Attribute	Value	% of Total
Genome size (bp)	5,031,571	100.00
DNA coding (bp)	4,482,414	89.00
DNA G + C (bp)	2,500,760	55.75
DNA scaffolds	1	100.00
Total genes	4,677	100.00
Protein coding genes	4,599	98.33
RNA genes	18	0.38
Genes with function prediction	3,356	71.75
Genes assigned to COGs	4,225	91.87
Genes with Pfam domains	4,406	94.20
Genes with signal peptides	1,605	37.99
CRISPR repeats	64	NA

**Table 4:** Number of genes associated with general COG functional categories

Code	Value	% age	Description
J	210	4.6	Translation, ribosomal structure and biogenesis
A	1	0	RNA processing and modification
K	397	8.6	Transcription
L	204	4.4	Replication, recombination and repair
B	7	0.2	Chromatin structure and dynamics
D	36	0.8	Cell cycle control, cell division, chromosome partitioning
V	64	1.4	Defense mechanisms
T	262	5.7	Signal transduction mechanisms
M	255	5.5	Cell wall/membrane biogenesis
N	114	2.5	Cell motility
U	88	1.9	Intracellular trafficking and secretion
O	178	3.9	Posttranslational modification, protein turnover, chaperones
C	300	6.5	Energy production and conversion
G	340	7.4	Carbohydrate transport and metabolism
E	518	11.3	Amino acid transport and metabolism
F	93	2.0	Nucleotide transport and metabolism
H	198	4.3	Coenzyme transport and metabolism
I	180	3.9	Lipid transport and metabolism
P	319	6.9	Inorganic ion transport and metabolism
Q	157	3.4	Secondary metabolites biosynthesis, transport and catabolism
R	626	13.6	General function prediction only
S	379	8.2	Function unknown
-	374	8.1	Not in COGs

The total is based on the total number of protein coding genes in the genome

## Insights from the genome sequence

BRIG [25] was used to generate the comparison between the fully-genome sequenced species *H. elongata*DSM 2581 ASM19687v1 (4.06 Mb, 63.6 % G + C) and *H. campaniensis* ASM69648v1 (4.07 Mb, 52.6 % G + C), and the draft sequence of the type strain *H. boliviensis* LC1 (4.2 Mb, 54.7 % GC). The comparison was performed on the nucleotide sequences with a lower cut off identity threshold of 50 %. The genome comparison reveals several unique regions in the *Halomonas* sp. R5-57 genome. Most of these include mobile genetic elements, and some contain genes for membrane transporters, secretion proteins and restriction-modification systems (Fig. 3a). *Halomonas* sp. R5-57 has the highest overall similarity to the recently deposited High-Quality Draft sequence of *Halomonas**sp*. TG39a (ASM74439v1; 4.9 Mb, 55.0 % G + C). A pairwise comparison using the nucleotide sequences of these two genomes and visualization in ACT [26] identified eight regions which differ between the two genomes: two of these appear to be translocations and correspond to parts of the *Halomonas* sp. R5-57 which are not found in *H. elongata*, *H. campaniensis*, or *H. boliviensis*, five others are insertions which are unique to *Halomonas* sp. R5-57 and one is an insertion in *Halomonas**sp*. TG39a Fig. 3b.

### Extended insights

Species of *Halomonas*, like other halotolerant chemorganotrophic bacteria, produce compatible solutes to maintain the osmotic balance inside their cells. An example is ectoine which is produced by cultivation of strains *H. boliviensis* and *H. elongata* [27]. The genes of *Halomonas* sp. R5-57 involved in ectoine biosynthesis, hydroxylation and transportation, as well as for the production of PHAs are listed in Table 5 together with their predicted properties and locus tags. The approximate position of these genes is shown on the graphical representation of the *Halomonas* sp. R5-57 chromosome (Fig. 3a). High homology is found between the two EctD protein products of *Halomonas* sp. R5-57 and *H. boliviensis* (89 % and 99 %) as well as their EctA, EctB, and Ect C sequences (98, 98 and 85 %). Homology is slightly lower between *Halomonas* sp. R5-57 and *H. elongata*: EctDs (69 % and 73 %) EctA (85 %), EctB (86 %), and Ect C (81 %).

**Table 5:** Genes from *Halomonas* sp. R5-57 predicted to be involved in production of ectoine and PHAs

Solute	Gene product	Function	Locus tag	MW (kDa)	pI
Ectoine	EctD	Ectoine hydroxylase	HALO0980	36.7	5.5
		5-carboxymethyl-2-hydroxymuconate delta-isomerase	HALO0981	24.1	4.8
	EctA	L-2,4-diaminobutyric acid acetyltransferase	HALO2492	21.1	5.0
	EctB	Diaminobutyrate-2-oxoglutarate transaminase	HALO2491	46.1	5.8
	EctC	Ectoine synthase	HALO2490	14.7	5.0
PHA	PHA B	acetoacetyl-CoA reductase	HALO4132	26.8	5.62
	PHA A	Acetyl-CoA acetyltransferase	HALO1910	41.0	6.0
	PHA A	Acetyl-CoA acetyltransferase	HALO2333	41.8	5.5
	PHA A	Acetyl-CoA acetyltransferase	HALO4196	40.5	5.6
	PHAC	PHB synthase	HALO2716	66.7	5.3
	PHAC	PHB synthase truncated	HALO3139 HALO3140	na	na
	PHAC	PHB synthase	HALO1802	71.8	4.9

PHAs are cellular energy-storage molecules that can serve as precursors for bioplastic production by humans, [12, 28]*.**Halomonas* sp. R5-57 carries three genes annotated as polyhydroxyalkanoate synthases (PHA Cs); the enzymes responsible for carrying out the final polymerization step in PHA biosynthesis [28]. The product of *phaC* HALO1802 has high homology with PHA C1sequences of *H. boliviensis* (91 %) and *H. campaniensis* (86 %) and with enzymes from *Halomonas* spp.O-1 (86 %) and *H. elongata* (77 %) which have recently been heterologously produced and characterized [29]. The putative PHA C (HALO2716) of *Halomonas* sp. R5-57 differs from the PHA C1 sequences, but has 75 % homology with another PHA C from *H. boliviensis*. A third possible PHA C comprising loci HALO3139 and HALO3140 contains a frameshift generating a stop codon after 67 amino acids, and is found within the phage-containing poorly-conserved 3367–3491 kbp region of the *Halomonas* sp. R5-57 genome (Fig. 3a). The *phaC* genes of *Halomonas* sp. R5-57 have been cloned, and their recombinant expression and structural elucidation is part of ongoing studies by our group to more fully understand the biochemical properties and catalytic mechanism of these enzymes.

Given its ability to tolerate salt concentrations up to 12 %, extracellular enzymes from *Halomonas* sp. R5-57 are expected to be functional under moderate-to-high salt conditions and thus could be employed in high-salinity reaction conditions. Functional screening of *Halomonas* sp. R5-57 using the API® system and plate-based assays revealed several secreted enzyme activities that could be of interest in industrial and biotechnological settings. Subsequent to genome sequencing, the genes annotated with enzyme classes that could impart these functions were identified together with putative signal peptides for secretion (Table 6).

**Table 6:** Enzyme activities detected by functional screening

Putative function (E. C. number)	Genes		Activity
	Total	Signal peptides	
Triacylglycerol lipase (3.1.1.3)	4	4	Lipase
Hydrolases acting on peptide bonds (protease, 3.4.-)	43 (20)	10	Gelatinase
Glycosidases hydrolysing O- and S-glycosyl compounds (3.2.1.-)	14	2	Chitinase
			Beta galactosidase
			Hydrolysis of esculin
Exodeoxyribonucleases (3.1.11.-)	6		DNAse
Endodeoxyribonucleases (3.1.21.-)	1		DNAse
Hydrolases acting on C-N bonds in linear amidines (3.5.3-)	7		Arginine dihydrolase
Nitrate reductases (1.7.99.4)	1		Nitrate reduction

A further possible application for *Halomonas* sp. R5-57 would be manipulation of its cellular machinery for use as a protein-expression host. The low-temperature and high-salinity growth optima could be potentially advantageous for recombinant production of psychrophilic or halophilic enzymes, which can suffer from poor solubility in commonly-used E. coli-based expression systems. Additionally, as osmolyte compounds are known to be potent protein stabilizers [30], their induction simultaneously with intracellular heterologous protein expression in *Halomonas* could present a further strategy to improve solubility of ‘difficult’ recombinant protein targets. The in-depth sequence information of halophilic bacterial strains, such as we have provided in this project will be key to engineering of such organisms in realization of this goal.

## Conclusions

*Halomonas* sp. R5-57 has several phenotypic and genetic features, which may impart useful properties in biotechnological applications. The complete genome sequence of *Halomonas* sp. R5-57 presented here will help utilization the biotechnological potential of this organism; either by whole-cell cultivation for production of high-value products such as ectoine and PHAs, or as a source of gene-mining for individual enzymes.

## Additional file

Additional file 1: Figure S1. Temperature and salinity optima of *Halomonas* sp. R5-57 grown in LB media. The growth rate represents the increase in absorbance at 600 nm during the exponential growth phase of cultures. (PNG 57 kb)

## Abbreviations

COG: Cluster of orthologous groups
PHAs: Polyhydroxyalkanoates
RDP: Ribosomal database project
SMRT: Single molecule real-time
