# Supplementary figures and images for: Complete genome sequence of Halomonas sp. R5-57
Source: Stand Genomic Sci. 2016 Sep 7;11(1):62. doi: 10.1186/s40793-016-0192-4 (PMC5015195; doi:10.1186/s40793-016-0192-4)

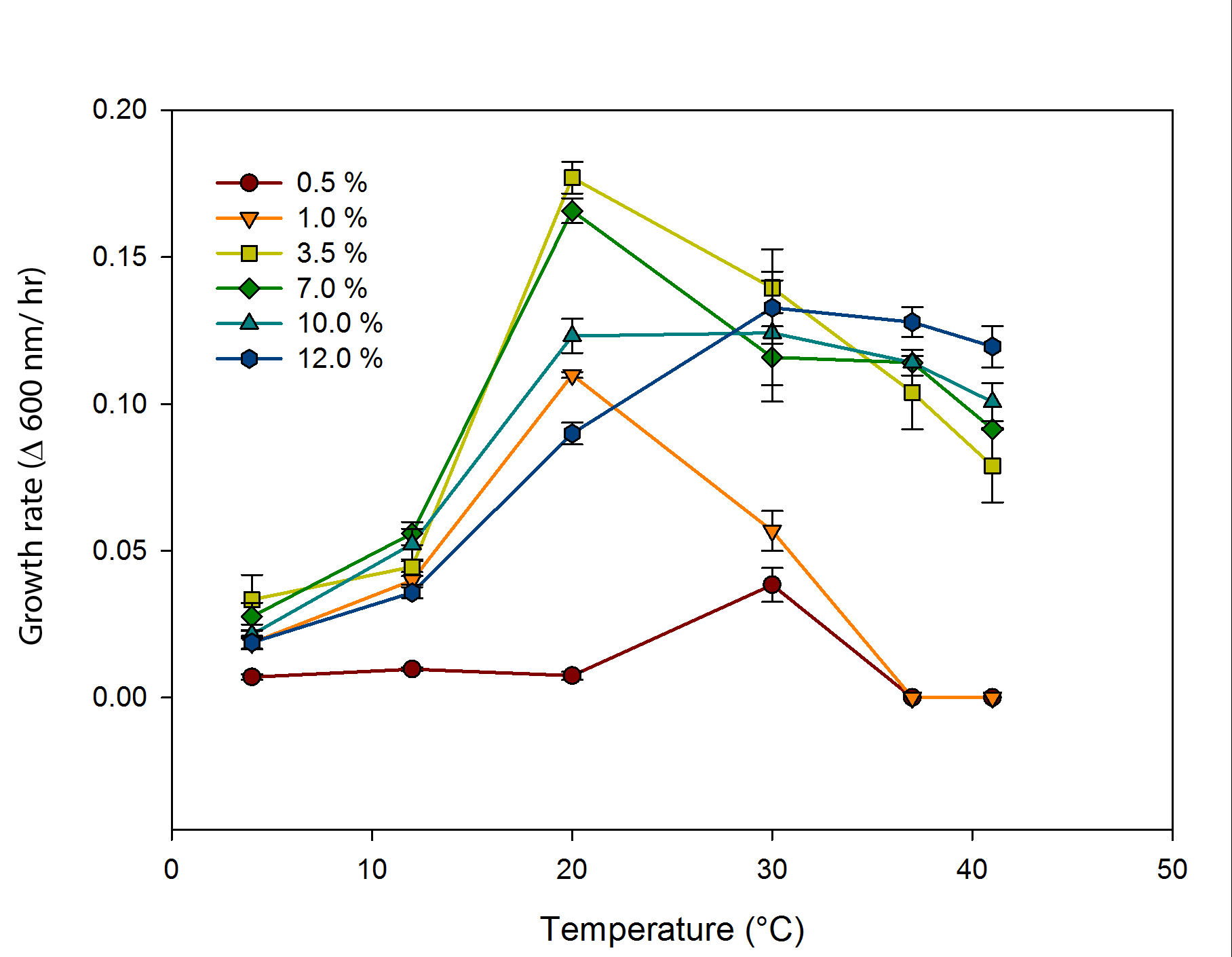

Supplement: Additional file 1: Figure S1. — Temperature and salinity optima of Halomonas sp. R5-57 grown in LB media. The growth rate represents the increase in absorbance at 600 nm during the exponential growth phase of cultures. (PNG 57 kb) [file 40793_2016_192_MOESM1_ESM.png]
